# The Inadequacy of Regulatory Frameworks in Time of Crisis and in Low-Resource Settings: Personal Protective Equipment and COVID-19

**DOI:** 10.1007/s12553-020-00429-2

**Published:** 2020-05-02

**Authors:** Leandro Pecchia, Davide Piaggio, Alessia Maccaro, Claudio Formisano, Ernesto Iadanza

**Affiliations:** 1 Applied Biomedical Signal Processing and Intelligent eHealth Lab, School of Engineering, University of Warwick, Coventry, UK; 2 Health Technology Assessment Division, International Federation for Medical and Biological Engineering, Florence, Italy; 3 Andrea Grimaldi” Medical Care, San Giorgio a Cremano, Naples, Italy; 4 Department of Information Engineering, University of Florence, Florence, Italy

**Keywords:** COVID-19, International standards, Personal protective equipment, Masks, Visors, Face-shields, CE marking

## Abstract

COVID-19 pandemic is plaguing the world and representing the most significant stress test for many national healthcare systems and services, since their foundation. The supply-chain disruption and the unprecedented request for intensive care unit (ICU) beds have created in Europe conditions typical of low-resources settings. This generated a remarkable race to find solutions for the prevention, treatment and management of this disease which is involving a large amount of people. Every day, new Do-It-Yourself (DIY) solutions regarding personal protective equipment and medical devices populate social media feeds. Many companies (e.g., automotive or textile) are converting their traditional production to manufacture the most needed equipment (e.g., respirators, face shields, ventilators etc.). In this chaotic scenario, policy makers, international and national standards bodies, along with the World Health Organization (WHO) and scientific societies are making a joint effort to increase global awareness and knowledge about the importance of respecting the relevant requirements to guarantee appropriate quality and safety for patients and healthcare workers. Nonetheless, ordinary procedures for testing and certification are currently questioned and empowered with fast-track pathways in order to speed-up the deployment of new solutions for COVID-19. This paper shares critical reflections on the current regulatory framework for the certification of personal protective equipment. We hope that these reflections may help readers in navigating the framework of regulations, norms and international standards relevant for key personal protective equipment, sharing a subset of tests that should be deemed essential even in a period of crisis.

## Introduction

As of early April 2020, the world is stricken by the recent pandemic outbreak [1] of a new strain of Coronavirus, previously unknown to mankind, denominated Severe Acute Respiratory Syndrome CoronaVirus 2 (SARS-CoV-2). This virus is part of the family of the coronaviruses, which are viruses commonly affecting mammals and birds. Although the respiratory tract infections on humans caused by this family of viruses are usually common colds (Human Coronavirus 229E, Human Coronavirus NL63, Human Coronavirus OC43, and Human Coronavirus HKU1), sometimes they beget viral pneumonia and rarely they can be the cause of a severe acute respiratory syndrome (SARS) (SARS-CoV, MERS-CoV, and SARS-CoV-2) [2]. SARS-CoV-2 causes a disease, better known as COVID-19 (CO for Corona, VI for Virus, D for disease and 19 for the year in which it was identified), with symptoms spanning from mild (e.g., fever, tiredness, dry and continuous cough, and shortness of breath, diarrhoea, and sore throat [3, 4]) to serious (e.g., viral pneumonia and multi-organ failure [5, 6]). SARS-CoV-2, similarly to other viruses [7], seems to have spilled over to humans from wild animals [8]. As a consequence, the human immune system, having never been in contact with such a virus, lacks the ability to fight against the pathogen [7], which can have particularly dangerous effects on subjects with already weak immune systems, or immunosuppressed or elderly subjects with existing preconditions. Based on the current information, the virus has been classed as a Hazard Group 3 (HG3) pathogen [9] and the World Health Organization (WHO) has stated that laboratory tests and practices should follow biosafety level 3 guidelines [10].

The COVID19 outbreak impact on European countries was twofold. First, the supply chain of personal protective equipment (PPE), medical devices (MDs), consumables and spare parts revealed its frailty in its dependence on China’s capability to produce them, severally hindered by the lockdown since January 2020. Second, this world pandemic has been causing an unprecedented demand of hospitalizations, especially in intensive care units (ICUs), since its early stages. This set off a chain reaction affecting a number of other routine hospitalizations (e.g., elective surgeries), which were postponed giving priority to ICU beds in terms of resources (spaces, personnel, equipment) Moreover, healthcare staff is highly exposed to the risk of catching COVID-19 themselves, due to the inner nature of their daily routine, which exposes them to physical contact with patients. The combination of these factors has created in Europe de facto conditions that are usually typical of ‘low-resource settings’, generating havoc among all the countries, independently from their wealth level.

The combination of a frail supply-chain and an unprecedented demand of ICU beds demonstrated the extent to which countries were not prepared to tackle global disasters, such as the current pandemic.

This is particularly evident in Europe, where many healthcare systems (e.g., France, Italy, Spain and the UK), being rated among the best ones in the world [11], are being heavily overburdened by the ever-increasing number of patients needing hospitalization or intensive care [12–15]. The national health systems, in fact, lack essential resources^1^ for dealing with COVID-19, including MDs (e.g. surgical masks, ventilators, infusion pumps), PPEs [16] and healthcare personnel (who is being reduced by the disease itself).

For the first time after decades, the progressive scarcity of devices, equipment and resources has raised also in high-income countries the problem of resource allocation and prioritisation. The latter could expose a part of the population, probably the most disadvantaged individuals, to further difficulties in accessing healthcare services [17].

The urgent need for equipment directly affects the role of clinical engineers, professionals who are in charge of verifying that all the medical and electro-medical devices are compliant with the essential requirements imposed by the national laws, before authorising their use in hospital settings. In Europe, this means compliance with the European framework of directives and regulations certified by the presence of the CE mark. Strictly following international standards is the regular path chosen by the manufacturers in order to guarantee the compliance of their products to the above-mentioned requirements, in terms of performances and safety. The current situation has highlighted the flaws of the regulations. For instance, the non-universality of regulations, norms and international standards is clearly evident in these situations of emergency. The problem of non-universality of technical norms is well-known in the context of low-resources settings, especially in the context of Low- and Middle-Income Countries (LMICs) as highlighted by the authors of this paper who have been extensively acting to overcome this issue [18, 19]. The COVID19 pandemic, is dramatically demonstrating that this limit is paramount also in high-income countries during emergencies. The international standards, indeed, proved to be often too generic and demanding, resulting difficult to be implemented in many countries, in terms of time, costs and overall effort required, thus jeopardizing a prompt response to emergencies. This is everyday evidence in lower-income countries, and it is becoming now clear also in high-income ones.

In this critical context, we have joined our efforts to write this manuscript in order to share our considerations on the necessity of identifying a set of minimum requirements to test PPEs for use in hospitals during the COVID19 pandemic. We hope this contribution may be relevant for the readers, helping them navigating the variegated context of PPEs regulatory framework. The proposed approach reflects a minimum set of tests that should always be considered despite the waivers issued by several states. This discussion should then be continued, once this crisis will be over, especially with regard to lower-income countries, where the inadequacy of international norms is clear also in everyday conditions.

## PPE regulation in EU and its inadequacy for COVID-19

The first European directive on the design, the manufacture and the marketing of PPEs was published in December 1989, exactly 30 years before SARS-CoV-2 was first identified in China (Directive 89/686/EEC). This Directive was superseded in 2016 by Regulation (EU) 2016/425, which outlines legal obligations in order to guarantee the highest quality and efficiency of the PPEs circulating in the EU internal market. The compliance with such regulation is assured by the CE marking. As per semantics, the change from directives to regulations was not trivial and implied a tougher and more harmonized approach. Directives, in fact, are EU instruments requiring member states to legislate accordingly and enforce them at a National level by relevant laws with a certain flexibility. Vice versa, EU Regulations are simultaneously enforced in each member state after a transitional period, imposing clear and detailed common rules, which do not give room for divergent transpositions by member states [20].

The Regulation (EU) 2016/425 itself refers to harmonized standards, developed by a recognized European Standards Organization (e.g., the European Committee for Standardization (CEN)) as a way in which manufacturers or Notified Bodies can assess the conformity of a product. As of Personal Eye Protection and Respiratory Protective Devices, EN166:2001 and EN 149:2001 + A1:2009 are the respective harmonized standards.

The pathway that manufacturers can take for achieving the CE Marking of PPE are different, depending on the PPE risk-category. For PPE used in low-risk conditions, manufacturers can rely on internal labs and tests to demonstrate that their products are compliant with the harmonised standards requirements, issuing self-certifications. When dealing with protection against SARS-CoV-2 virus, the PPEs are framed in the highest risk class (class II or III), requiring the involvement of external notified bodies. Notified Bodies are appointed under the Article 28 of Regulation (EU) 2016/425, and their responsibilities include confirming that PPE have an adequate level of health and safety in accordance with the essential requirements laid down for that product in harmonised standards. According to the number of PPE produced, Notified Bodies may be requested to repeat this test several times per year. This may include testing few samples that manufactures send to the Notified Body, as well as inspections in the manufacturing farms. A key point is that there is a series of tests that need to be performed according to the relevant standards, depending on the type of device and equipment, to prove that the designed solutions fulfil the minimum requirements in terms of performance and safety. This series of tests is not specific for hospital settings, but it is general, aiming at including all the working conditions to which PPE could be exposed, including testing the robustness of PPE after having been exposed to extreme temperatures (e.g., 70 °C), which are never reached in hospitals due to harmonised standards for medical locations.

Therefore, when dealing with general standards, their generality becomes a too stringent constraint in emergency situations. For example, PPEs such as face shields or particle filtering face masks (e.g., FFP2 or FFP3 masks) are also (and more often) required in settings other than hospitals, such as in carpentry or soldering activities, in conditions very different from the ones needed by hospital settings. The tests required by the international norms reflect these extreme working conditions, which are different from the ones that can be found in hospitals. Some national standards developing organisations and some notified bodies, aware of the fact that requiring redundant tests is not strictly necessary in conditions of emergency, reconsidered the whole procedure for testing PPEs and reduced it to a subset of essential tests and minimum requirements that should be met for the use in a COVID-19 hospital department. A similar approach to the one described by Badnjevic et al. [21] can be used for standardised procedures with minimum requirements for assessing medical equipment in healthcare settings in situations of crisis and emergency.

Confirming this criticism, on the 13th March 2020 the EU Commission published the Recommendation EU 2020/403 (non-legislative act), providing guidance for conformity assessment and market surveillance procedures within the context of the COVID-19 threat. This recommendation has proved the resiliency of the European Commission and its outstanding capability to react to crisis, while confirming the inadequacy of European Regulation for crises and scarce resource scenarios.

Amid this widespread confusion, the scientific community has to make clear the fact that, especially during a crisis, PPE should meet the highest possible quality standards, in accordance with the ALARP (As Low As Reasonably Possible) risk management principle [22].

## Amatorial solutions and the importance of international standards and CE marking

The importance of meeting high-quality standards, guaranteeing the efficiency of medical devices and equipment and the safety of their users, is evident now more than ever. During this pandemic, we are witnessing growing proliferation of amatorial initiatives (i.e., quick fixes) aiming at providing for the above-mentioned needs. These initiatives, amplified through social media, although certainly driven by good intentions, may beget a series of solutions potentially as harmful as the problem they are trying to solve, if not properly mentored. The innumerable solutions one can come across on the internet span from using baking paper to reproduce a (surgical!) face mask to 3D printing respirators using cotton filters, claiming that they are effective in filtering the virus. The lack of any risk assessment, albeit minimal, poses major risks for the user. Using materials from vacuum cleaner filters to realize filtering face masks, for example, could be a threat for the user’s safety, in case of presence of dangerous glass microfibers [23].

Also in this context, regulations and standards are essential, as they sum up the state of the art, resulting from a series of field experiences, aimed at guiding the manufacturer in designing and producing devices with high levels of safety. In this regard, visual inspections performed by experienced technicians are the first approaches that can be used to evaluate if the obtained prototype is safe-by-design. For example, even a small abrasion or defect due to sub-optimal materials or inappropriate manufacturing processes, can potentially lead to discomfort and skin irritation or lesions in the long run. This possibility, potentially dangerous in normal working conditions, is even more risky in extraordinary working conditions. In severe working conditions (due to extended/more frequent shifts for lack of personnel and to stressful conditions), mistakes or distractions are even more likely. A robust design is thought to be resilient to these conditions as well.

### The review of standards in situations of emergency – A case study in UK

The UK Office for Product Safety and Standards (OPSS) has been working with manufacturers to understand where regulatory requirements were preventing them from delivering the products the public and NHS needed, offering continuously updates and guidance.

The 10^th^ of April 2020, the OPSS' position is that manufacturers can deploy PPE provided that:

- it meets the essential safety requirements, and
- conformity assessment procedures have been started via a Notified Body, even if the conformity assessment, “including affixing of CE marking procedures”, has not been completed.

The OPSS has also issued an official letter to UK Notified Bodies providing further guidance on the European Commission Recommendation (EU 2020/403) and urging them to prioritise the conformity assessment of PPEs necessary for protection in the context of the COVID-19 and requesting this work to be conducted swiftly. With this letter, notified bodies were also allowed to test the products using other than harmonised standards, nominally World Health Organisation recommendations.

UK Notified Bodies reacted promptly prioritising such tests, aiming at assessing essential safety requirements specifically thinking to NHS workers during the COVID-19 pandemic. As a result, PPEs can now be tested much faster (e.g., in 1 week for face shields), for one third of the usual cost circa and with also a reduced number of samples to be sent for the tests. Certainly, this has been a prompt and functional response. However, once this COVID crisis will be overcome, there should be a wider discussion on how to make norms and regulations intrinsically more flexible so as to always allow rapid interventions to ever-changing situations and to be inclusive of many different specificities. This could potentially be achieved either by including some default waivers which could allow modifications in case of need, or by reformulating the way the regulations are conceived, basing the new ones on a better-defined and effective universality.

#### Face shields

Face shields (aka visors) are part of a family of devices named personal eye protective equipment, as defined in EN 166:2001. According to EN ISO 4007:2018, the term “protector” (3.5.1.1) for purposes of eye and face protection includes eye protectors (3.5.1.2), eye guards (3.5.1.4), face protectors (3.5.1.5), face screens/shields (3.5.1.6) and more (i.e. goggles, hand shields, protective masks, spectacles, visors).

As afore mentioned EN 166 provides for a series of tests for the evaluation of face shields that are inclusive of any possible future use, including the industrial environment in which the worker is at high risk of injuring his/her eyes and/or face with splinters or electric arcs. However, to respond to the urgent needs related to the COVID-19, national standards bodies have reduced the number and type of essential tests to be performed on face shields to be used in healthcare settings for COVID-19. This fits perfectly the concept of norms as technical means to reach the state of the art. Technical standards are never mandatory. The authors of this paper have been in touch with different international experts and standards bodies investigating what type of test they would carry out on PPE in this situation of emergency. Based on these discussions and on the subset of tests individuated by the standards bodies, the authors of this paper drafted their own subset of suggested tests. Table 1 summarizes the tests that the authors of this paper believe to be essential for face shields that are to be used as an additional protection by healthcare workers in this pandemic. EN 166 refers to EN 167:2002 for some test specifications; however, EN167 was recently superseded by EN ISO 18526:2020. Therefore, the authors created Table 1 inserting the links to the correct clauses in the new standard, where applicable.

**Table 1:** The subset of tests (EN 166) deemed essential to evaluate personal eye protection equipment for COVID-19

Performance requirement	Test method clause	Requirement
General construction (Section 6.1 EN 166:2002)	Visual inspection and manufacturer’s certificates	Eye-protectors shall be free from projections, sharp edges or other defects which are likely to cause discomfort or injury during use.
Materials (6.2)	Visual inspection and manufacturer’s certificates	No parts of the eye-protector which are in contact with the wearer shall be made of materials which are known to cause any skin irritation.
Headbands (6.3)	By measuring	Headbands, when used as the principal means of retention, shall be at least 10 mm wide over any portion which may come into contact with the wearer’s head. Headbands shall be adjustable or self-adjusting.
Field of vision (7.1.1)	The size of the field of vision is defined in conjunction with the appropriate head-form described in clause 17 of EN 168:2001. The test shall be carried out in accordance with clause 18 of EN 168:2001.	Eye-protectors shall exhibit a minimum field of vision defined by the two ellipses when placed and centered at a distance of 25 mm from the surface of the eyes of the appropriate head-form. The horizontal axis shall be parallel to and 0,7 mm below the height of the line connecting the centres of the two eyes.
		The horizontal length of the ellipses shall be of 22.0 mm, the vertical width of the ellipses shall be 20.0 mm. The centre distance of the two ellipses shall be d = c + 6 mm, where c is the pupillary distance. The pupillary distance is 64 mm for the medium head-form and 54 mm for the small head-form, if not specified differently by the manufacture.
Spherical, astigmatic and prismatic refractive powers (7.1.2.1)	The refractive powers of oculars shall be measured by the reference methods specified in clause 6 of EN ISO 18526-1:2020. If, during measurement using the telescope, a doubling or other aberration of the image is observed, then the test sample shall be subjected to further examination using the test method described in 6.3 of EN ISO 18526-1:2020	The permissible tolerances for oculars without corrective effect are given in the Clause 7.1.2.1.2 of EN166.
Transmittance – oculars without filtering action (7.1.2.2–1)	Clause 6 of EN ISO 18526-2:2020	Oculars intended to protect the eyes against mechanical or chemical hazards only, and cover plates, shall have a luminous transmittance greater than 74.4%.
Diffusion of light (7.1.2.3)	Clause 14 of EN ISO 18526-2:2020	The max value of the reduced luminance factor shall be 0.50 cd/(m^2^*lx) for all the other oculars.
Quality of material and surface (7.1.3)	Clause 6.6 of EN ISO 18526-3:2020	Except for a marginal area 5 mm wide, oculars shall be free from any significant defects likely to impair vision in use, such as bubbles, scratches, inclusions, dull spots, pitting, mould marks, scouring, grains, pocking, scaling and undulation.
Minimum robustness (7.1.4.1)	Clause 3.1 of EN 168:2001	The 7.1.4.1 requires performing an impact test with a 22 mm nominal diameter steel ball, impacting with a force of (100 ± 2) N.
		On so testing the following defects shall not occur:
		a) ocular fracture: an ocular shall be considered to have fractured if it cracks through its entire thickness into two or more pieces, or if more than 5 mg of the ocular material becomes detached from the surface away from the one in contact with the ball, or if the ball passes through the ocular;
		b) ocular deformation: an ocular shall be considered to have been deformed if a mark appears on the white paper on the opposite side to the one on which the force is applied.
		Note: in period of crisis such as the COVID, this should be at least tested using realistic simulations
Resistance to corrosion (7.1.6)	Clause 6.9 of EN ISO 18526-3:2020	After the test, all metal parts of the eye-protector shall display smooth surfaces, free from corrosion, when they are examined by a trained observer.
		Note: This should be extended to plastic parts considering the sterilization processes and substances (e.g., after cleaning visibility should not be compromised)
Resistance to ignition (7.1.7)	Clause 6.10 of EN ISO 18526-3:2020	Eye-protectors shall be considered to be satisfactory if no part of the eye-protector ignites or continues to glow after removal of the steel rod.
Protection against (droplets) and splashes of liquid (7.2.4)	Clause 12 of EN 168:2001	Face-shields cover the eye-region rectangle of the appropriate head-form as described in 10.2.2.2 of EN 168:2001 as assessed in accordance with 10.2 of EN 168:2001. Additionally, face-shields for protection against splashes of liquids shall have a viewing area with a minimum vertical centre-line depth of 150 mm when mounted in the appropriate housing.
Lateral protection (7.2.8)	Clause 6.4 of EN ISO 18526-3:2020	Eye-protectors claimed to provide lateral protection shall pass the lateral region coverage assessment
Resistance to fogging (7.3.2)	Clause 6.11 of EN ISO 18526-3:2020	If oculars are described as resistant to fogging they shall remain free from fogging for a minimum of 8 s when tested.

#### Filtering half masks

Filtering half masks to protect against particles are part of a family of equipment named “respiratory protective devices”, which are regulated by EN 149:2001 + A1:2009. They are defined as respiratory protective devices that consist substantially of filter material or comprise a facepiece in which the main filter(s) form an inseparable part of the device. These devices can be provided with valves for inspiration and exhalation. Depending on their filtering power, they can be designated as FFP1, FFP2 and FFP3. FFP2 and FFP3 half masks, which are suggested as a protection against COVID-19 [24], belong to category III and require, for CE marking, a certificate from and external notified body, issued after exams on the product technical documentation and tests on the product itself, as stated in the European Regulation 2016/425.

As per the face shields, also the tests enlisted for these devices are designed taking into account all the most disparate conditions of use. The authors of this paper, after having discussed with different national standards bodies, similarly to their approach with face shields, summarised in Table 2 the tests that they believe to be essential for this piece of equipment to be used by healthcare workers in this pandemic, beyond the biocompatibility evaluation.

**Table 2:** The subset of tests (EN 149:2001 + A1:2009) deemed essential to evaluate respiratory protective devices for COVID-19

Performance requirement	Test method clause	Requirement
Visual inspection (Section 7.3 EN 149:2001 + A1:2009)	The visual inspection is carried out where appropriate by the test house prior to laboratory or practical performance tests (Clause 8.2).	
Packaging (7.4)	The visual inspection is carried out where appropriate by the test house prior to laboratory or practical performance tests (Clause 8.2).	Particle filtering half masks shall be offered for sale packaged in such a way that they are protected against mechanical damage and contamination before use.
Cleaning and disinfecting (7.6)	Testing shall be done in accordance with Clause 8.4 and Clause 8.5. With reference to 7.9.2, after cleaning and disinfecting the re-usable particle filtering half mask shall satisfy the penetration requirement of the relevant class. Testing shall be done in accordance with Clause 8.11.	After cleaning and disinfecting the re-usable particle filtering half mask shall satisfy the penetration requirement of the relevant class.
If the particle filtering half mask is designed to be re-usable, the materials used shall withstand the cleaning and disinfecting agents and procedures to be specified by the manufacturer.		
Practical performance (7.7) The particle filtering half mask shall undergo practical performance tests under realistic conditions.	2 particle filtering half masks shall be tested as received in accordance with Clause 8.4.	During the tests the particle filtering half mask shall be subjectively assessed by the wearer and after the test, comments on the following shall be recorded:
		a) head harness comfort;
These general tests serve the purpose of checking the equipment for imperfections that cannot be determined by the tests described elsewhere in this standard.		
		b) security of fastenings;
		c) field of vision;
		d) any other comments reported by the wearer on request.
Where practical performance tests show the apparatus has imperfections related to wearer’s acceptance, the test house shall provide full details of those parts of the practical performance tests which revealed these imperfections.		
Total inward leakage (7.9.1) The laboratory tests shall indicate that the particle filtering half mask can be used by the wearer to protect with high probability against the potential hazard to be expected. The total inward leakage consists of three components: face seal leakage, exhalation valve leakage (if exhalation valve fitted) and filter penetration.	At least 5 specimens shall be tested as received in accordance with Clause 8.5.	All samples must achieve the specifics.
		All individual exercise results tests shall be not greater than 11% (FFP2) (5% for FPP3) and, in addition, all arithmetic means for the total inward leakage shall be not greater than 8% (FFP2) (2% for FFP3).
Penetration of filter material (7.9.2)	At least 3 samples shall be tested as received for NaCl and paraffin oil (PO) for 3 min in accordance with Clause 8.11.	The maximum penetration of test aerosol shall be: • 6% for both PO and NaCl for FFP2 • 1% for both PO and NaCl for FFP3
Carbon dioxide content of the inhalation air (7.12)	At least 3 particle filtering half masks shall be tested in accordance with Clause 8.7.	The carbon dioxide content of the inhalation air (dead space) shall not exceed an average of 1.0% (by volume).
Head harness (7.13)	Testing shall be done in accordance with Clause 8.4 and 8.5.	The head harness shall be designed so that the particle filtering half mask can be donned and removed easily. The head harness shall be adjustable or self-adjusting and shall be sufficiently robust to hold the particle filtering half mask firmly in position and be capable of maintaining total inward leakage requirements for the device.
Field of vision (7.14)	Testing shall be done in accordance with Clause 8.4.	The field of vision is acceptable if determined so in practical performance tests.
Breathing resistance (7.16)	At least 3 samples shall be tested as received in accordance with Clause 8.9.	The maximum permitted resistances per volumetric flow rate for FFP2 are:
		• 30 l/min – 0.7 mbar (inhale)
		• 95 l/min – 2.4 mbar (inhale)
		• 160 l/min – 3.0 mbar (exhale)
		For FFP3 are:
		• 30 l/min – 1.0 mbar (inhale)
		• 95 l/min – 3.0 mbar (inhale)
		• 160 l/min – 3.0 mbar (exhale)

## Similarities between the COVID-19 pandemic and low-resource settings

COVID-19 pandemic and low-resource settings have deep differences, but also many commonalities. Low-resource settings are common in LMICs, but also exist in rural and remote areas of many high-income ones. Contrary to the common belief, the main problem of low-resource settings lays beyond the lack of funding. Several studies have in fact highlighted the fact that if this had been the main problem, donations would have solved it [19, 25]. Conversely, modern medicine requires much more than budget, as it is evident especially in the remit of medical devices and equipment, included PPE. In fact, low-resource settings are often characterised by the lack of clinical knowledge, lack of specialised clinical personnel and technical staff, scarcity of medical devices, drugs and spare parts due to a jeopardised supply-chain [19, 26]. As argued above, COVID-19 has simultaneously hindered the supply-chain, increased the ICU hospitalization demand and reduced staff. This created de facto conditions that are quite common in low-resource settings, especially in LMICs.

The COVID-19 pandemic demonstrated the lack of knowledge and lack of preparedness of many high-income countries, aggravated by the slowness in perceiving the complexity of the situation faced by other countries, affected by the COVID-19 months in advance. For instance, before facing the COVID-19 outbreak in Milan metropolitan area, Italian authorities and experts of virology failed to understand the complexity of the situation in China and failed to acknowledge the great work done by Chinese colleagues. The same inertia has affected many north European Countries, which failed to appreciate the complexity of the COVID-19 disaster in Italy, despite the prompt response of Regional Institutions, especially in the South. While we write this paper, it seems that the USA reaction is again demonstrating some degree of inertia, failing again to acknowledge the severity of the situation in many European Countries, despite the effort that those countries are putting into the limitation of this pandemic.

Despite the deep differences, the shift of methodology from low-resource settings to the COVID-19 pandemic response may help speeding-up the response to the emergency, also in high-income countries. After all, shifting from domains where we lack solutions to a domain where we have established methods and tools has been one of the most powerful engineering solutions. An example could be the application of transformations such as Laplace transform to differential problems, bringing them in a domain where the equations become algebraic and can be easily solved in analytic form, and then shifted in the original domain using an anti-transform.

## Conclusion

The COVID19 outbreak has shown clearly the unsuitability of PPEs’ regulatory framework, body of norms, and international standards to extreme conditions. This was evident to the professionals working in low-resource settings, such as low- and middle-income countries and it emerged now powerfully also for high-income countries during the COVID-19 pandemic. The European regulatory framework evolved in the 1990s, mainly to protect European manufacturers from the unsustainable competition from manufacturers producing abroad. This evolution has been also driven by the manufacturers’ need to produce PPEs for the widest market possible, therefore following the principle of generalism (i.e., PPE tested to be used in any context) as opposed to particularism (i.e., PPE tested to be used in a specific context, such as nurses working in hospital wards). The prevalence of generalism over particularism resulted in a loss of universality, and in the fact that norms that can be sustained in normal conditions, at least by high-income countries, become unsustainable in times of crisis. These norms, which are often assumed as standards de facto also in many non-EU countries (e.g. in many African countries), are clearly not sufficiently universal for the contexts of low- and middle-income countries.

In this manuscript, two examples of simplified protocols starting from existing harmonized norms are presented and discussed. Similar exemplifications are currently accepted by European notified bodies in some EU Countries and could guide the realisation of tests in low- and middle-income countries. Starting from this unprecedented crisis, high-income countries will have to reconsider the nature of this regulatory framework and of these norms and international standards. The main lessons that the biomedical and clinical engineering community should learn from this terrible experience is that there is a major need for an evidence-based regulatory framework, responding to the need of lead and lay users, rather than those of the market itself.

## Footnotes

1 https://www.who.int/emergencies/diseases/novel-coronavirus-2019/technical-guidance/covid-19-critical-items
